# Supplementary material for: Unequal segregation of mitochondria during asymmetric cell division contributes to cell fate divergence in sister cells in vivo
Source: Nat Commun. 2025 Aug 4;16:7174. doi: 10.1038/s41467-025-62484-5 (PMC12322191; doi:10.1038/s41467-025-62484-5)
Supplement: Supplementary file 1 — Supplementary Information [file 41467_2025_62484_MOESM1_ESM.pdf]

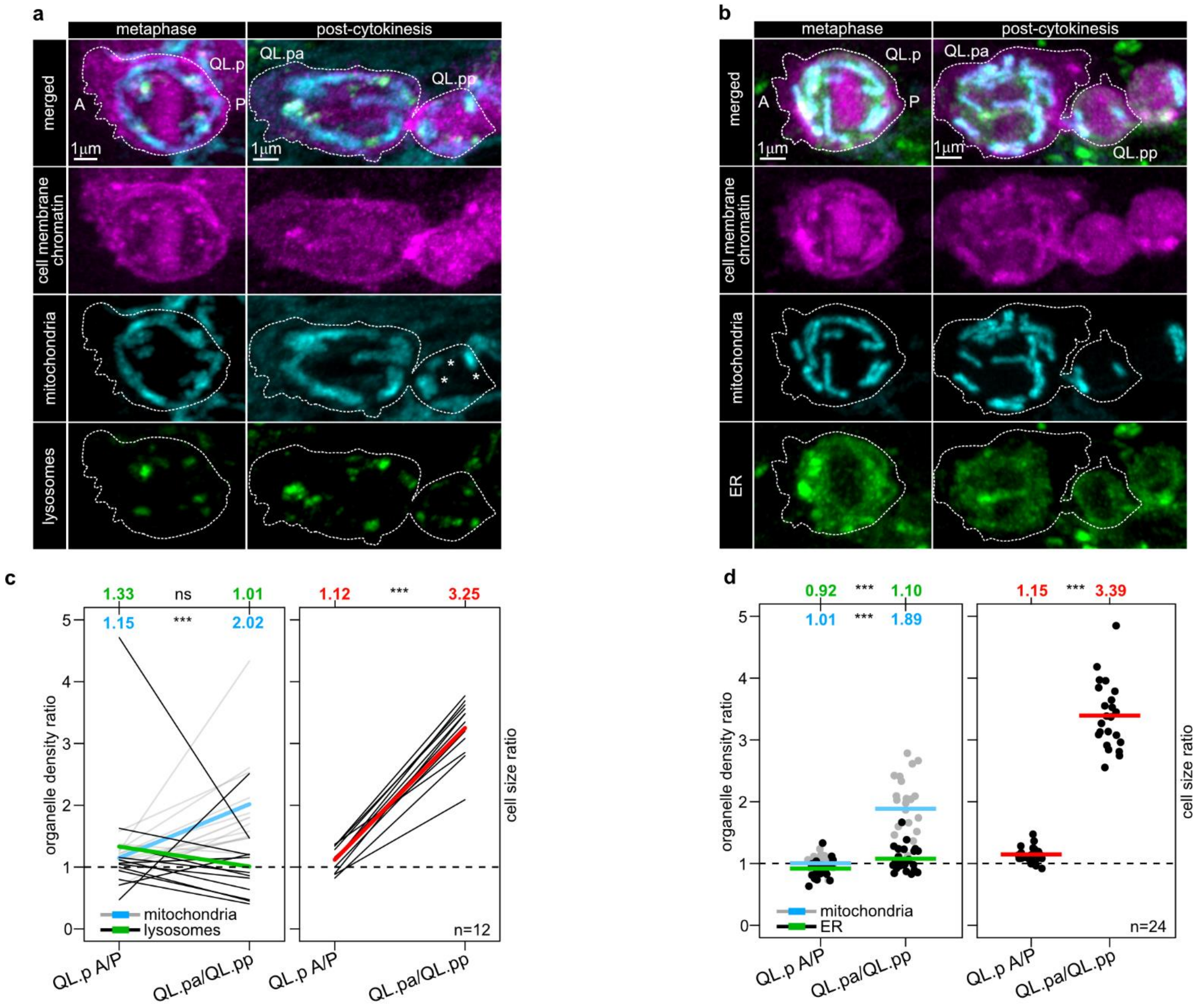


**Figure S1. Partitioning of lysosomes and ER during QL.p division.** **a**) Representative images of QL.p at metaphase and post-cytokinesis with chromatin (SFmTurquoise2ox::his-24) and cell membrane (myristoylated SFmTurquoise2ox) labelled in magenta, mitochondria (tomm-20::mKate2) in cyan, and lysosomes (eyfp::cup-5) in green (see main text for further information). **b**) Representative images of QL.p at metaphase and post-cytokinesis with chromatin (SFmTurquoise2ox::his-24) and cell membrane (myristoylated SFmTurquoise2ox) labelled in magenta, mitochondria in cyan (mtGFP), and ER (SP12-mCherry-KDEL) in green (see main text for further information). **c, d**) Analysis of organelles density ratio at metaphase and post-cytokinesis (left), and cell size ratio (right). Measurements of organelles and cell size are based on the mean integrated density and the area (μm$^2$) on each section of z-stacks, respectively, and not on volume. P values are calculated using the two-sided Wilcoxon matched pairs signed rank test (**c**, left; lysosomes V=57, p=0.176; mitochondria V=1 p <0.001), the two-sided paired t-test (**c**, right; cell size t(11)=-17.49, p<0.001), the two-sided unpaired t-test (**d**, left (mitochondria; t(24)=8.175, p<0.001), and **d**, right; cell size t(24)=-19.491, p<0.001), and the Mann-Whitney test (**d**, left (ER; W=6, p<0.001)). Normality was tested with the Shapiro-Wilk test. *: P value ≤ 0.05; **: P value ≤ 0.01; ***: P value ≤ 0.001; ****: P value ≤ 0.0001. Red, cyan and green lines (and respective numbers) = average (**c, d**). Black and grey dots and lines represent data from individual QL.p divisions (**c, d**). Data in panels **a, c** are derived from animals expressing the

*bcIs159* or *bcIs162* transgenes, respectively, while data in panels **b, d** are derived from animals expressing the transgene *bcIs160*. n= 12 and n=24 in **c** and **d**, respectively. Source data are provided as a Source Data file.

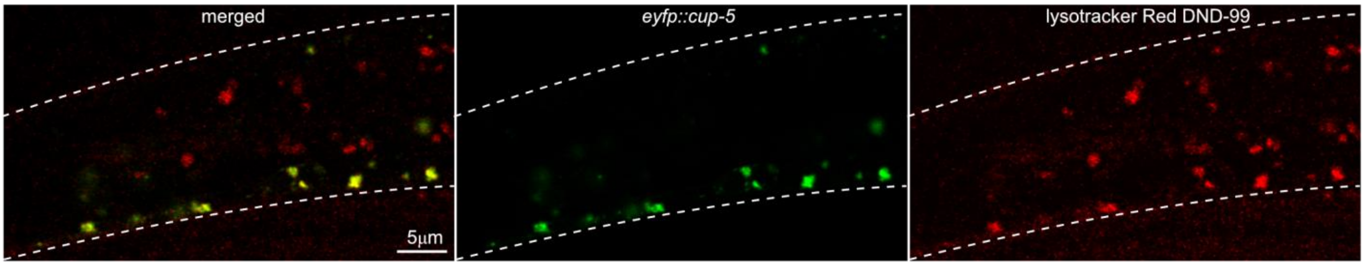


**Figure S2. *eyfp::cup-5* transgene labels specifically lysosomes.** Colocalization of *eyfp::cup-5* reporter expression with lysotracker Red DND-99 in the posterior side of an L1 larva expressing the *bcIs159* transgene. This *eyfp::cup-5* transgene labels lysosomes in some cells, but not all, because its expression is driven by *mab-5* promoter, which is active only in those respective cells, among which posterior Q neuroblasts.

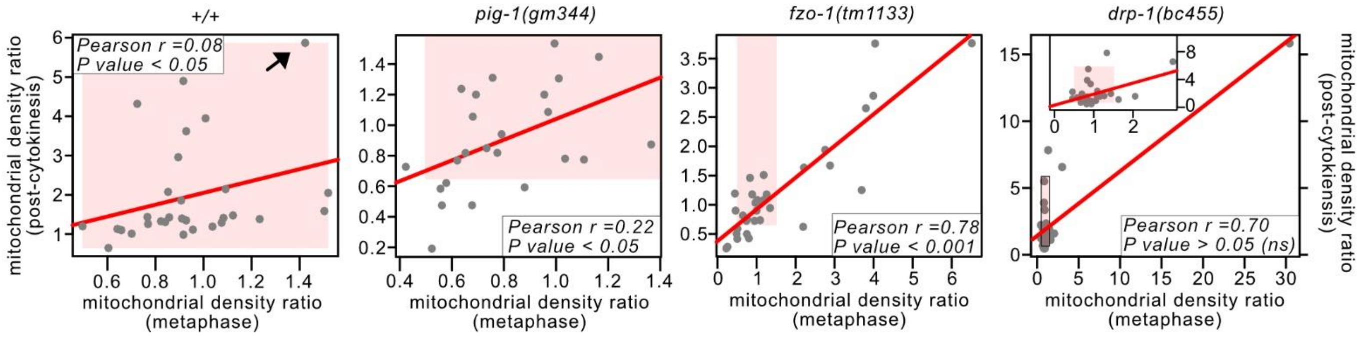


**Figure S3. Correlation between QL.p mitochondrial density ratios at metaphase (QL.pa A/P) and post-cytokinesis (QL.pa/QL.pp).** Red lines = linear regression fitted to scatter plots (dots represent values from individual QL.p divisions). Pearson's or Spearman's tests for association between samples were performed where appropriate, indicated by the coefficient of determination (r2) or the square of Spearman's rho (ρ2) are respectively given, illustrating the proportion of shared variance. Note that the correlation in wild type (+/+) shows a slope and data distribution similar to that in the respective correlation plot in Fig. 1b (bottom left corner). However, the correlation in the current figure (Fig. S3, +/+) is significant due to the data point on the top-right corner (black arrow). Removal of this outlier results in the loss of significance. Normality was tested using the Shapiro-Wilk test, and homoscedasticity was tested using Bartlett's test. In all genotypes, animals express the *bcIs153* transgene. Source data are provided as a Source Data file.

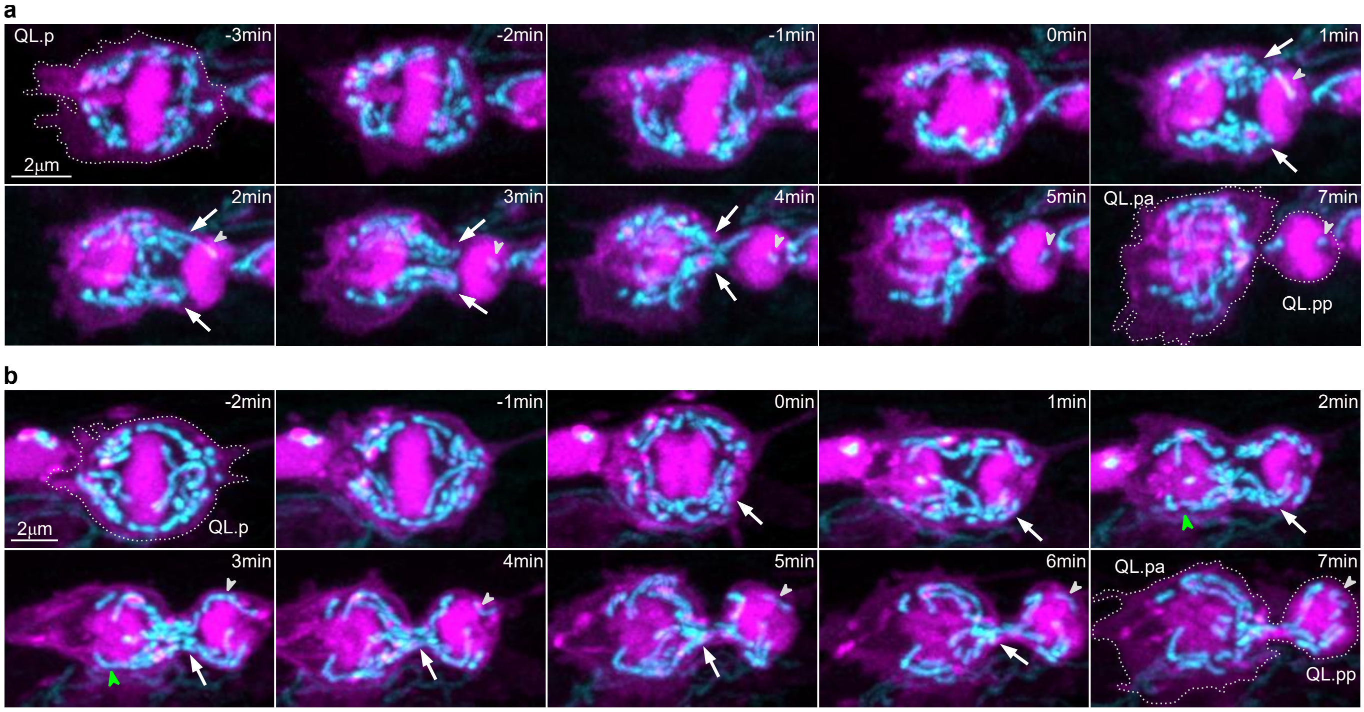


**Figure S4. Super-resolution timeseries of mitochondrial segregation during QL.p division in wild-type animals.** Super-resolution live two-colour time series of QL.p division. Plasma membrane (myristoylated mCherry) and chromatin (mCherry::his-24) are showed in magenta, mitochondria (mtGFP) in cyan (*bcIs153* transgene). Images are maximum intensity projections of aligned z-stacks. In all images, anterior is left and posterior is right. From top to bottom: wildtype examples representative of higher (**a**), and lower (**b**) mitochondrial density ratio (QL.pa/QL.pp). Arrows point to anteriorly directed transport of mitochondria. Green and white arrowheads point to mitochondrial fusion and fission, respectively. These timeseries are referred to in Fig. 2.

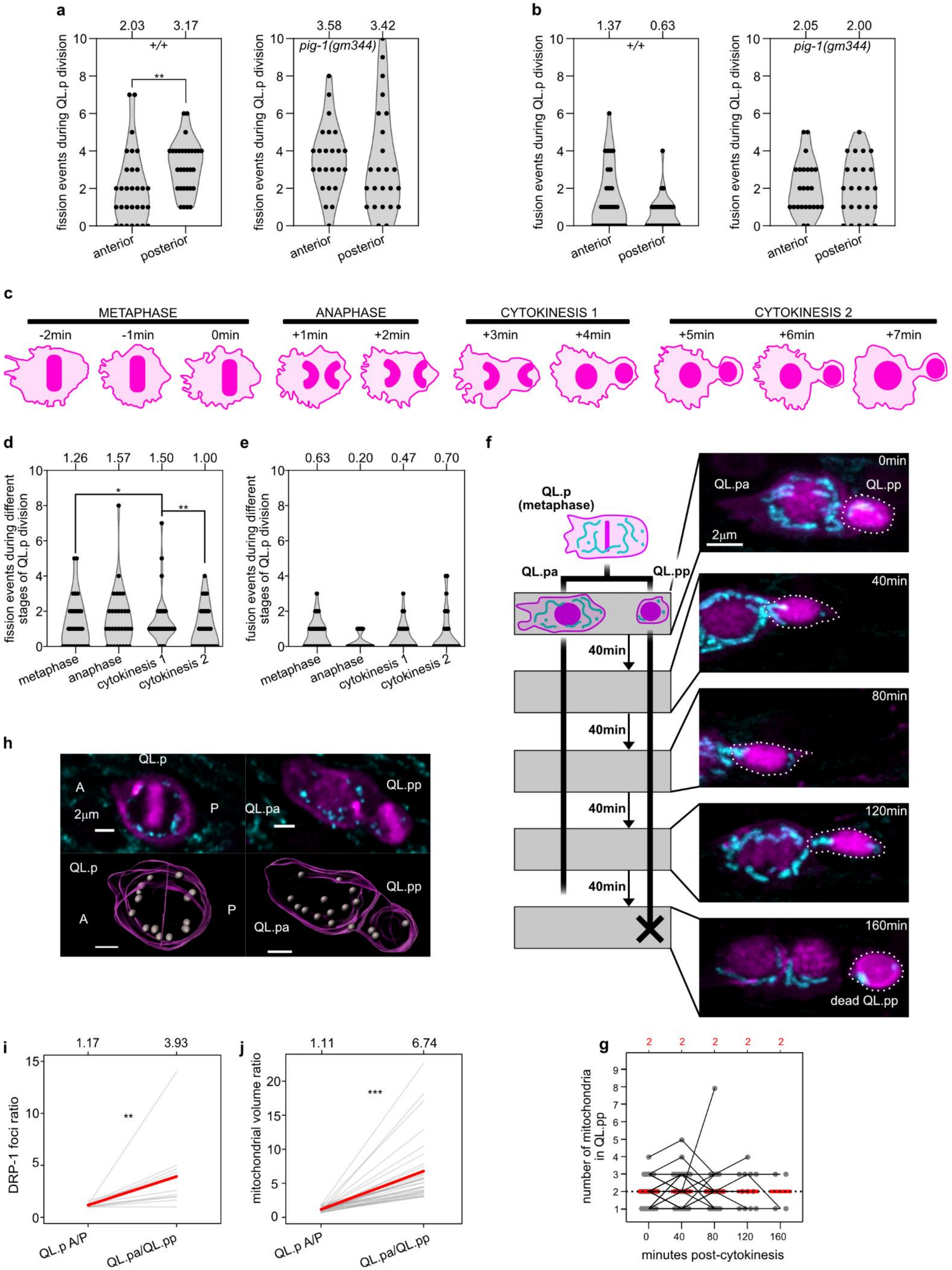
a
+/+
pig-1(gm344)
fission events during QL.p division
anterior
posterior
b
fusion events during QL.p division
c
METAPHASE
ANAPHASE
CYTOKINESIS 1
CYTOKINESIS 2
d
fission events during different stages of QL.p division
e
fusion events during different stages of QL.p division
metaphase
anaphase
cytokinesis 1
cytokinesis 2
f
QL.p (metaphase)
QL.pa
QL.pp
40min
dead QL.pp
h
A
P
2μm
i
DRP-1 foci ratio
QL.p A/P
QL.pa/QL.pp
j
mitochondrial volume ratio
g
number of mitochondria in QL.pp
minutes post-cytokinesis

**Figure S5. Mitochondrial fission and fusion events during QL.p division**. Recordings were visually inspected for fission and fusion events throughout the sections of individual z-stacks. **a**, **b**) Mitochondrial fission (**a**) and mitochondrial fusion (**b**) events per division in the anterior and posterior sides of QL.p between metaphase and post-cytokinesis in wild type (+/+) and *pig-1(gm344)* animals expressing the *bcIs153* transgene. Wild type fission χ2(7, N = 60) p = 0.0146, wild type fusion χ2(5, N = 60) p = 0.2873 , pig-1(gm344) fission χ2(10, N = 48) p = 0.4924, pig-1(gm344) fusion χ2(5, N = 48) p = 0.4070.**c**) Definition of QL.p division stages (metaphase, anaphase, cytokinesis 1, and cytokinesis 2). **d, e**) Fission (**d**) and fusion (**e**) events at different stages of division in wild type. **d**) metaphase-anaphase χ2(6, N = 60) p = 0.5067, metaphase-cytokinesis1 χ2(6, N = 60) p = 0.0357 , metaphase-cytokinesis2 χ2(5, N = 60) p = 0.6623, anaphase-cytokinesis1 χ2(7, N = 60) p = 0.1443, anaphase-cytokinesis2 χ2(5, N = 60) p = 0.6443, cytokinesis1-cytokinesis2 χ2(6, N = 60) p = 0.0218. **e**) metaphase-anaphase χ2(3, N = 60) p = 0.0858, metaphase-cytokinesis1 χ2(3, N = 60) p = 0.7593 , metaphase-cytokinesis2 χ2(4, N = 58) p = 0.4389, anaphase-cytokinesis1 χ2(3, N = 50) p = 0.3286, anaphase-cytokinesis2 χ2(4, N = 58) p = 0.2052, cytokinesis1-cytokinesis2 χ2(4, N = 58) p = 0.7400. **f**) Schematics of the imaging strategy of QL.pp during its survival time, between post-cytokinesis and QL.pp death, in wild-type animals. The grey boxes highlight super-resolution acquisitions of QL.pp, every 40 minutes starting at the post-cytokinesis time point. The time series is representative of 21 QL.pp survival times imaged from 21 animals. **g**) Number of mitochondria in QL.pp at different times between QL.p division (0min) and cell death (between 80 and 160 minutes after cytokinesis). time0-time40 χ2(4, N = 40) p = 0.7914, time0-time80 χ2(3, N = 42) p = 0.7742 , time0-time120 χ2(3, N = 33) p = 0.8075, time0-time160 χ2(1, N = 25) p = 0.4240. n=21. **h**) Average intensity projection of endogenous DRP-1(*dx230*Internal) foci (cyan) with mCherry-marked membrane and chromatin (magenta; transgene *rdvIs1*) recordings at metaphase (left) and post-cytokinesis (right), including respective whole 3D renderings in Imaris. **i**) DRP-1 foci ratio (anterior/posterior) during QL.p division in *drp-1(dx191)* (V=44, p=0.365). **j**) Mitochondrial volume ratio (anterior/posterior) during QL.p division in wild type (+/+; transgene *bcIs153*) (t(29)=-6.400, p<0.001). Red lines and red numbers represent the median for **g**, and the mean for **i-j**. Statistical significances shown represent χ2tests for **a, b, d, e,** and **g**; paired t-tests for **j**; and exact Wilcoxon-Pratt signed-rank test for **i**. Sample sizes are 30 and 24 for +/+ and *pig-1(gm344)*, respectively, in **a, b, d** and **e**. Sample size is 11 in **i**, 30 in **j**, and 21 in **g**. *: P value ≤ 0.05; **: P value ≤ 0.01; ***: P value ≤ 0.001. Source data are provided as a Source Data file.

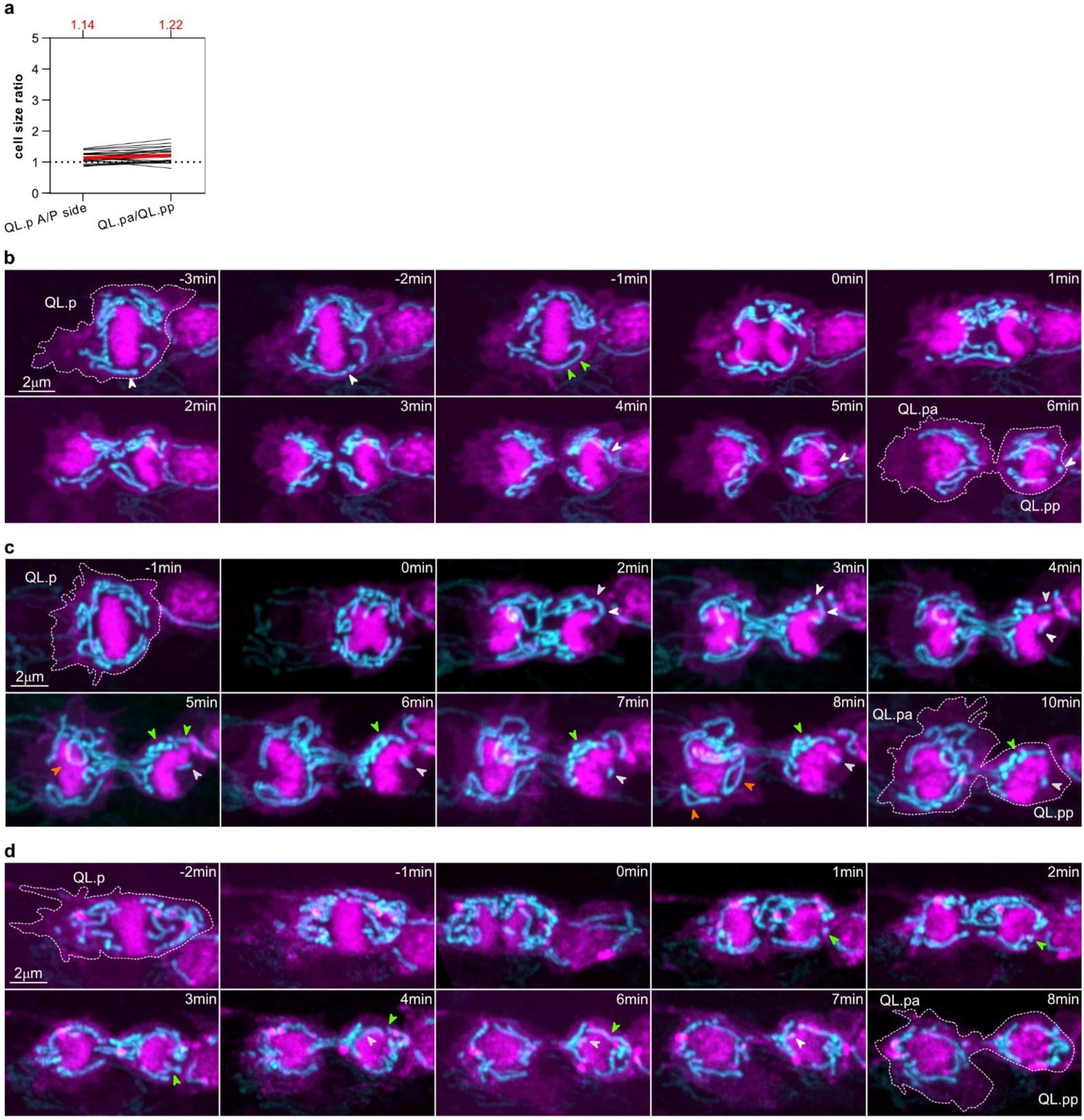


**Figure S6. Super-resolution timeseries of mitochondrial segregation during QL.p division in *pig-1(lf)* animals. a)** Cell volume ratio before (QL.p A/P sides) and after division (QL.pa/QL.pp) in *pig-1(gm344)* animals (t(23) = 2.051 p = 0.0518) (two-sided t test) (n=25)**. b-d**) Super-resolution live two-colour time series of QL.p division. Plasma membrane (myristoylated mCherry) and chromatin (mCherry::his-24) are showed in magenta, mitochondria (mtGFP) in cyan (*bcIs153* transgene). Images are maximum intensity projections of aligned z-stacks. In all images, anterior is left and posterior is right. From top to bottom: *pig-1(gm344)* examples representative of higher (**b**), average (**c**), and lower (**d**) mitochondrial density ratio (QL.pa/QL.pp). Green and white arrowheads point to mitochondrial fusion and fission, respectively. These timeseries are referred to in Fig. 2. Source data are provided as a Source Data file.

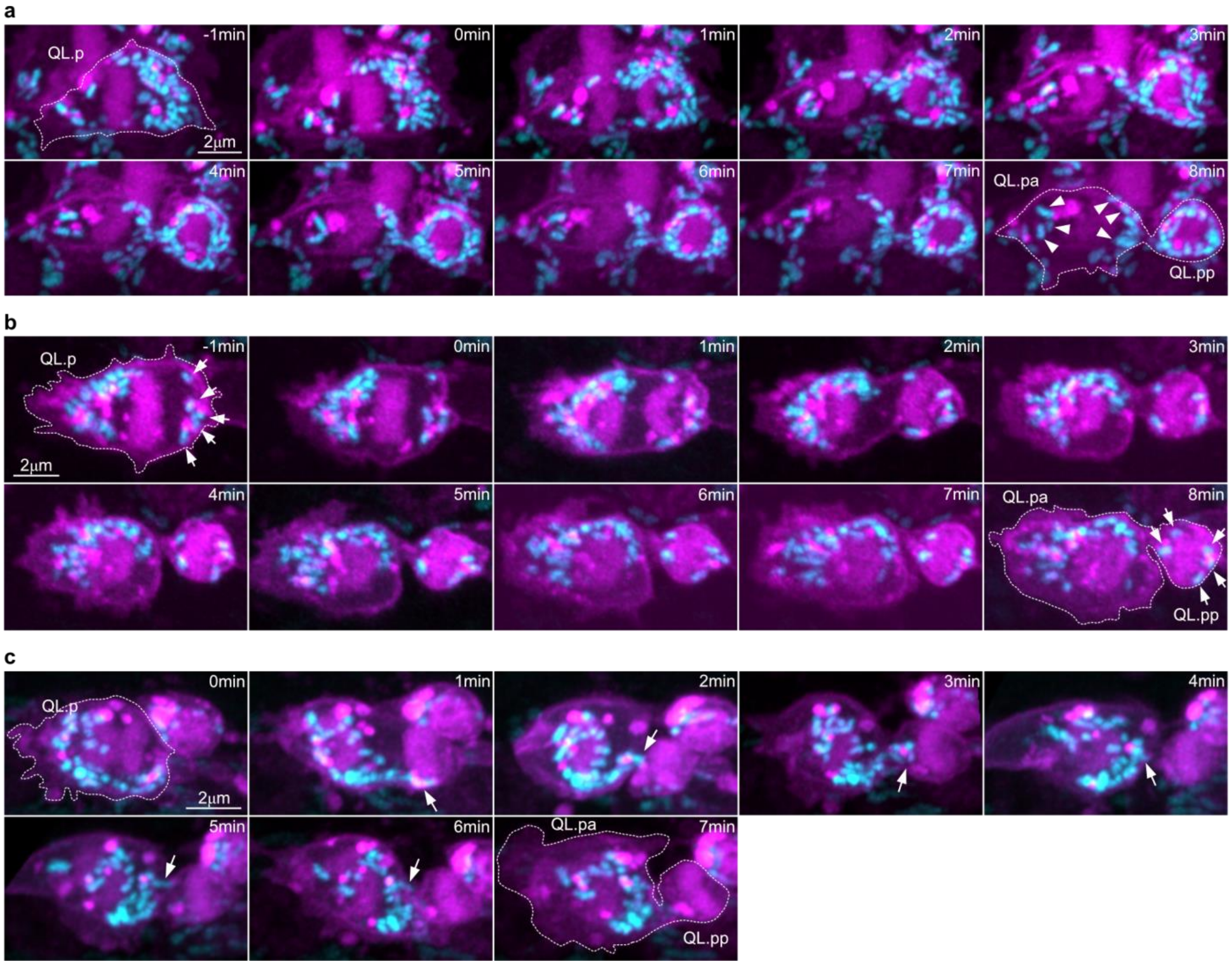


**Figure S7. Super-resolution timeseries of mitochondrial segregation during QL.p division in *fzo-1(lf)* animals.** Super-resolution live two-colour time series of QL.p division. Plasma membrane (myristoylated mCherry) and chromatin (mCherry::his-24) are showed in magenta, mitochondria (mtGFP) in cyan (*bcIs153* transgene). Images are maximum intensity projections of aligned z-stacks. In all images, anterior is left and posterior is right. From top to bottom: *fzo-1(tm1133)* examples representative of lower (**a**), average (**b**), and higher (+∞) (**c**) mitochondrial density ratio (QL.pa/QL.pp). Arrowheads point to QL.pa mitochondria (**a**). Arrows point to posterior mitochondria (metaphase) that were all inherited by QL.pp (**b**) and to the anteriorly directed movement of mitochondria (**c**). These timeseries are referred to in Fig. 2. The example in panel **c** is not showed in Fig. 2.

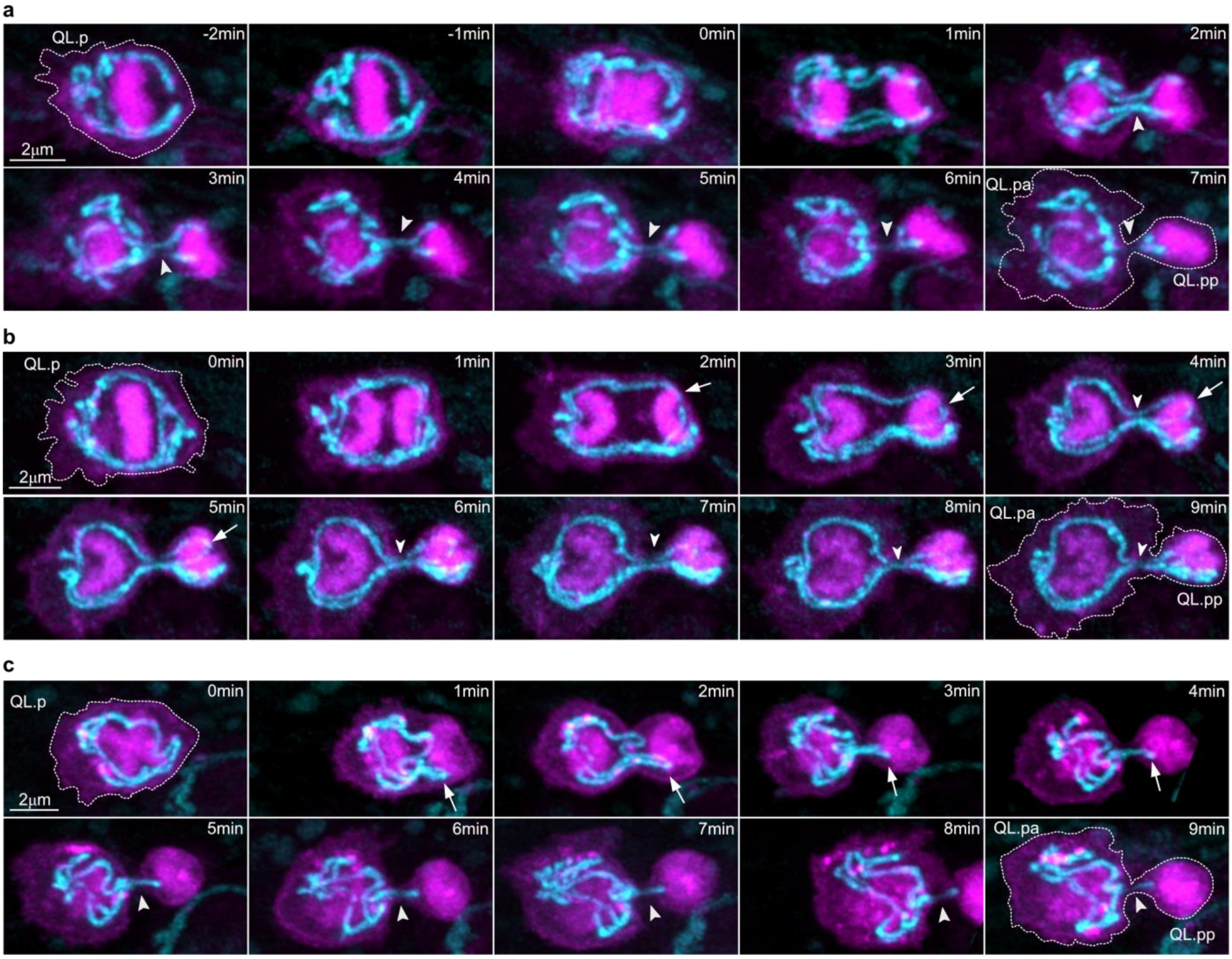


**Figure S8. Super-resolution timeseries of mitochondrial segregation during QL.p division in *drp-1(lf)* animals.** Super-resolution live two-colour time series of QL.p division in animals expressing *bcIs153*. Plasma membrane (myristoylated mCherry) and chromatin (mCherry::his-24) are showed in magenta, mitochondria (mtGFP) in cyan. Images are maximum intensity projections of aligned z-stacks. In all images, anterior is left and posterior is right. Top (**a**) and centre (**b**): *drp-1(bc455)* examples representative of average and lower mitochondrial density ratio (QL.pa/QL.pp). Bottom (**c**): *drp-1(tm1108)* example representative of higher mitochondrial density ratio (QL.pa/QL.pp) (see main text for further information). Arrowheads point to mitochondrial fission, whereas arrows point to a posterior mitochondrial portion looped around the posterior chromosome set blocking the anterior movement of mitochondria (**b**) and to the anteriorly directed movement of mitochondria (**c**). These timeseries are referred to in Fig. 2.

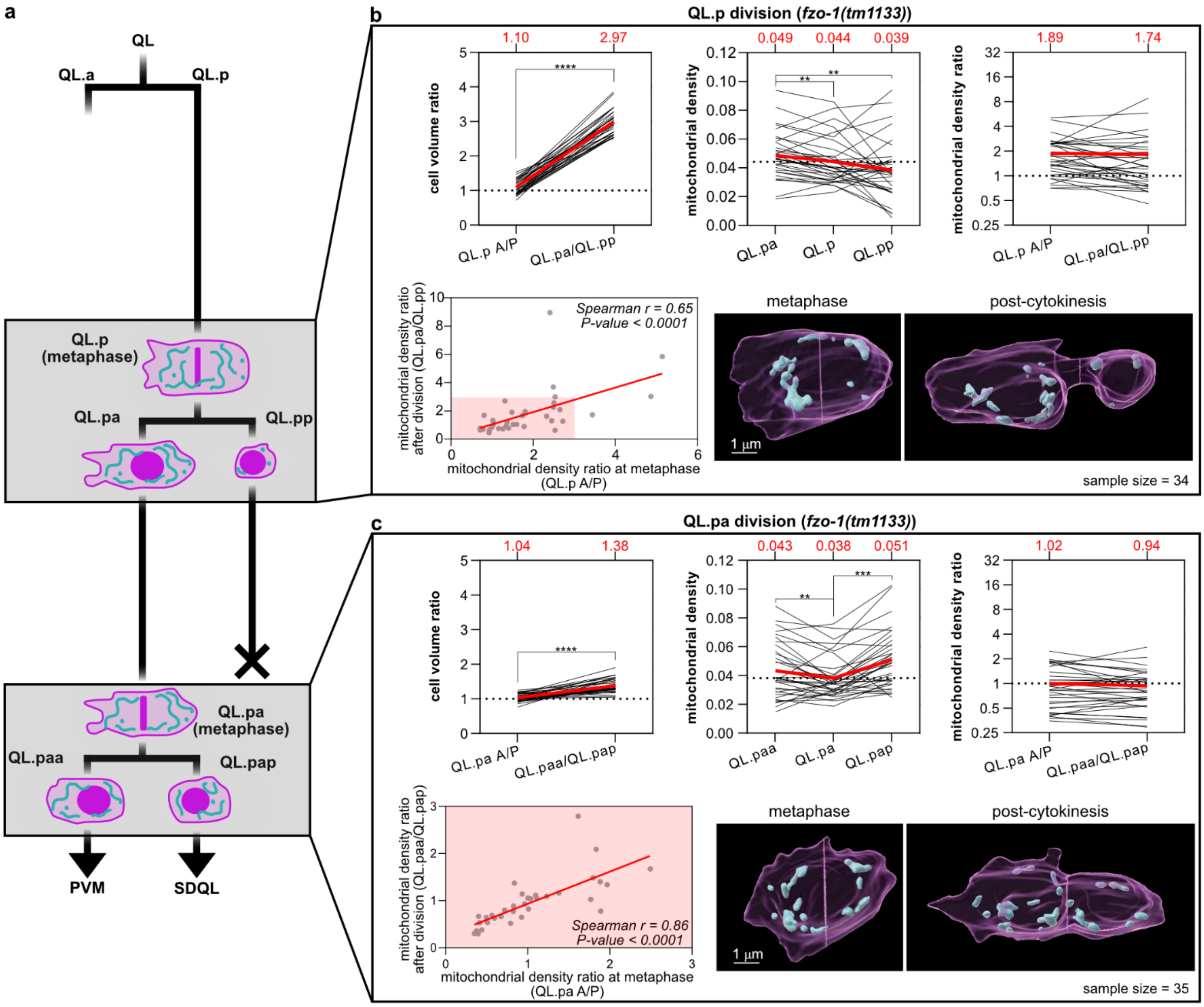


**Figure S9. Mitochondrial partitioning in *fzo-1(lf)* mutants. a**) Schematics of the QL.p lineage recorded in *fzo-1(tm1133)* animals expressing the *bcIs153* transgene. The grey boxes highlight both QL.p and QL.pa divisions. **b**) Measurements of cell volume ratio, mitochondrial density and mitochondrial density ratio during QL.p division. Top left: cell volume ratio before (QL.p A/P sides) and after division (QL.pa/QL.pp); top centre: mitochondrial density before (QL.p) and after division (QL.pa and QL.pp); top right: mitochondrial density ratio before (QL.p A/P sides) and after division (QL.pa/QL.pp); bottom left: correlation between mitochondrial density ratio before (x axes) and after (y axes) division. Cell volume ratio W = 595.0 $p < 0.0001$, QL.pa-QL.p Q(34) = 0.0040 p = 0.0053, QL.pa-QL.pp Q(34) = 0.0040 p = 0.0076, QL.p-QL.pp t(34) = 0.3162 p = 0.9035, mitochondrial density ratio W = -191.0 p = 0.1049. **c**) Measurements of cell volume ratio, mitochondrial density and mitochondrial density ratio during QL.pa division. Top left: cell volume ratio before (QL.pa A/P sides) and after division (QL.paa/QL.pap); top centre: mitochondrial density before (QL.pa) and after division (QL.paa and QL.pap); top right: mitochondrial density ratio before (QL.pa A/P sides) and after division (QL.paa/QL.pap); bottom left: correlation between mitochondrial density ratio before (x axes) and after (y axes) division; bottom right: representative 3D volumes of QL.pa division. Cell volume ratio t(34) = 8.453 $p < 0.0001$, QL.paa-QL.pa Q(35) = 0.0015 p = 0.0028, QL.paa-QL.pap Q(35) = 0.4010 p = 0.4028, QL.pa-QL.pap Q(35) = 0.0001 p = 0.0001, mitochondrial density ratio W = -140.0 p = 0.2585. P values are calculated using the Wilcoxon

matched pairs signed rank test (both **b** and **c**, top right), the paired t-test (both **b** and **c**, top left), the RM one-way ANOVA with FDR correction (**b**, top centre), the Friedman test with FDR correction (**c**, top centre), and the Spearman correlation (both **b** and **c**, bottom left). Normality was tested with the Shapiro-Wilk test. *: P value ≤ 0.05; **: P value ≤ 0.01; ***: P value ≤ 0.001; ****: P value ≤ 0.0001. Red lines = mean (both **b** and **c**, top row). In the top row plots of panels **a** and **b**, individual black lines represent the trends of each division between metaphase and post-cytokinesis or between QL.p and QL.pa and QL.pp. Red lines and red numbers = average (both **b** and **c**, top row). n=35 in **a** and **b**. Source data are provided as a Source Data file.

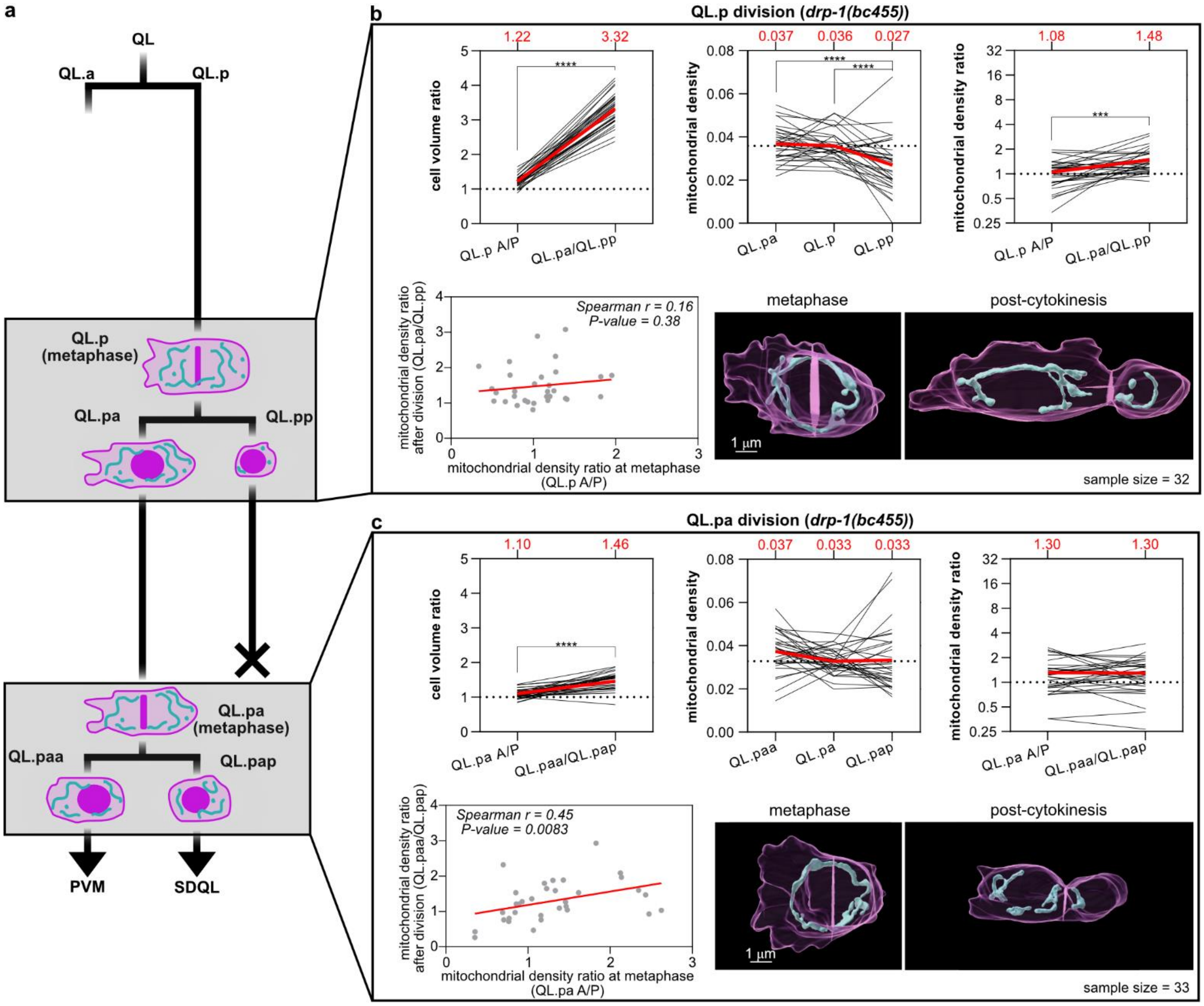


**Figure S10. Mitochondrial partitioning in *drp-1(lf)* mutants. a**) Schematics of the QL.p lineage recorded in *drp-1(bc455)* animals expressing the *bcIs153* transgene. The grey boxes highlight both QL.p and QL.pa divisions. **b, c**) Equivalent to Fig. S9a, b in both the arrangement and the content of plots. **b**) Cell volume ratio $t(32) = 28.04$ $p < 0.0001$, QL.pa-QL.p $t(32) = 0.7044$ $p = 0.1702$, QL.pa-QL.pp $t(32) = 5.536$ $p < 0.0001$, QL.p-QL.pp $t(32) = 4.371$ $p < 0.0001$, mitochondrial density ratio $W = 352.0$ $p = 0.0006$. **c**) Cell volume ratio $t(32) = 8.292$ $p < 0.0001$, QL.paa-QL.pa $Q(33) = 0.0770$ $p = 0.0489$, QL.paa-QL.pap $Q(33) = 0.0770$ $p = 0.0364$, QL.pa-QL.pap $Q(33) = 0.9471$ $p = 0.9020$, mitochondrial density ratio $W = 29.0$ $p = 0.8050$. P values are calculated using the Wilcoxon matched pairs signed rank test (both **b** and **c**, top right), the paired t-test (both **b** and **c**, top left), the RM one-way ANOVA with FDR correction (**b**, top centre), the Friedman test with FDR correction (**c**, top centre), and the Spearman correlation (both **b** and **c**, bottom left). Normality was tested with the Shapiro-Wilk test. *: P value ≤ 0.05; **: P value ≤ 0.01; ***: P value ≤ 0.001; ****: P value ≤ 0.0001. Red lines = mean (both **b** and **c**, top row). In the top row plots of panels **a** and **b**, individual black lines represent the trends of each division between metaphase and post-cytokinesis or between QL.p and QL.pa and QL.pp. Red lines and red numbers = average (both **b** and **c**, top row). n=35 in **a** and **b**. Source data are provided as a Source Data file.

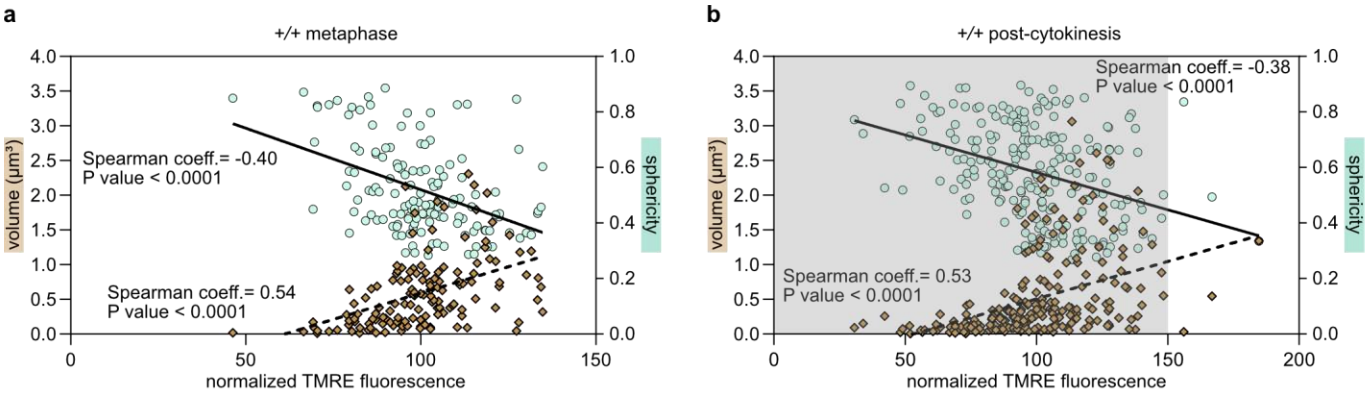


**Figure S11. Mitochondrial activity (TMRE) and mitochondrial morphology correlate.** Correlation between normalized TMRE fluorescence and mitochondrial sphericity and volume at single mitochondrion level at metaphase (**a**) and post-cytokinesis (**b**), in wildtype animals expressing the *bcIs158* transgene. The mean TMRE intensity within each mitochondrion was normalized by dividing it with the mean TMRE intensity of all mitochondria in the respective cell (QL.p for metaphase or QL.pa and QL.pp, together, for post-cytokinesis) (see methods for further information). The grey panel in **b** refers to the XY dimensions of in **a**. Continuous line= linear regression of the TMRE-mitochondrial sphericity correlation; dotted lines= linear regression of TMRE-mitochondrial volume correlation. Source data are provided as a Source Data file.

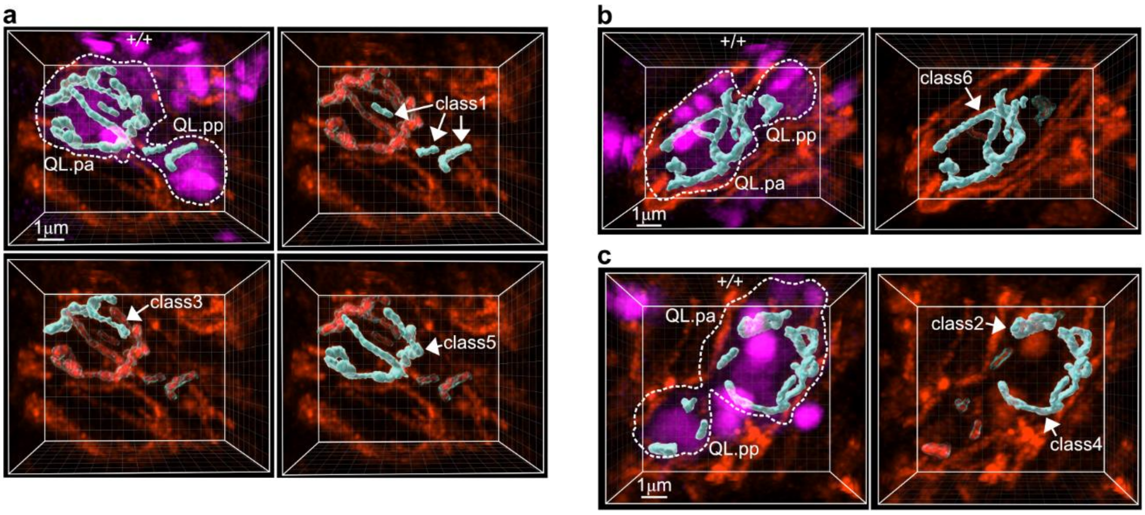


**Figure S12. Mitochondrial volume classes.** Representative images showing examples of the six volume classes of mitochondria used to study TMRE fluorescence intensity in relation to mitochondrial morphology (mitochondria are bigger and longer and/or branched going from classes 1 to 6) in animals expressing the *bcIs158* transgene. The six classes span equally the wild-type volume range 0.0043μm$^3$-3.0620 μm$^3$. **a**) mitochondrial classes 1,3 and 5; **b**) mitochondrial class 6; **c**) mitochondrial classes 2 and 4.

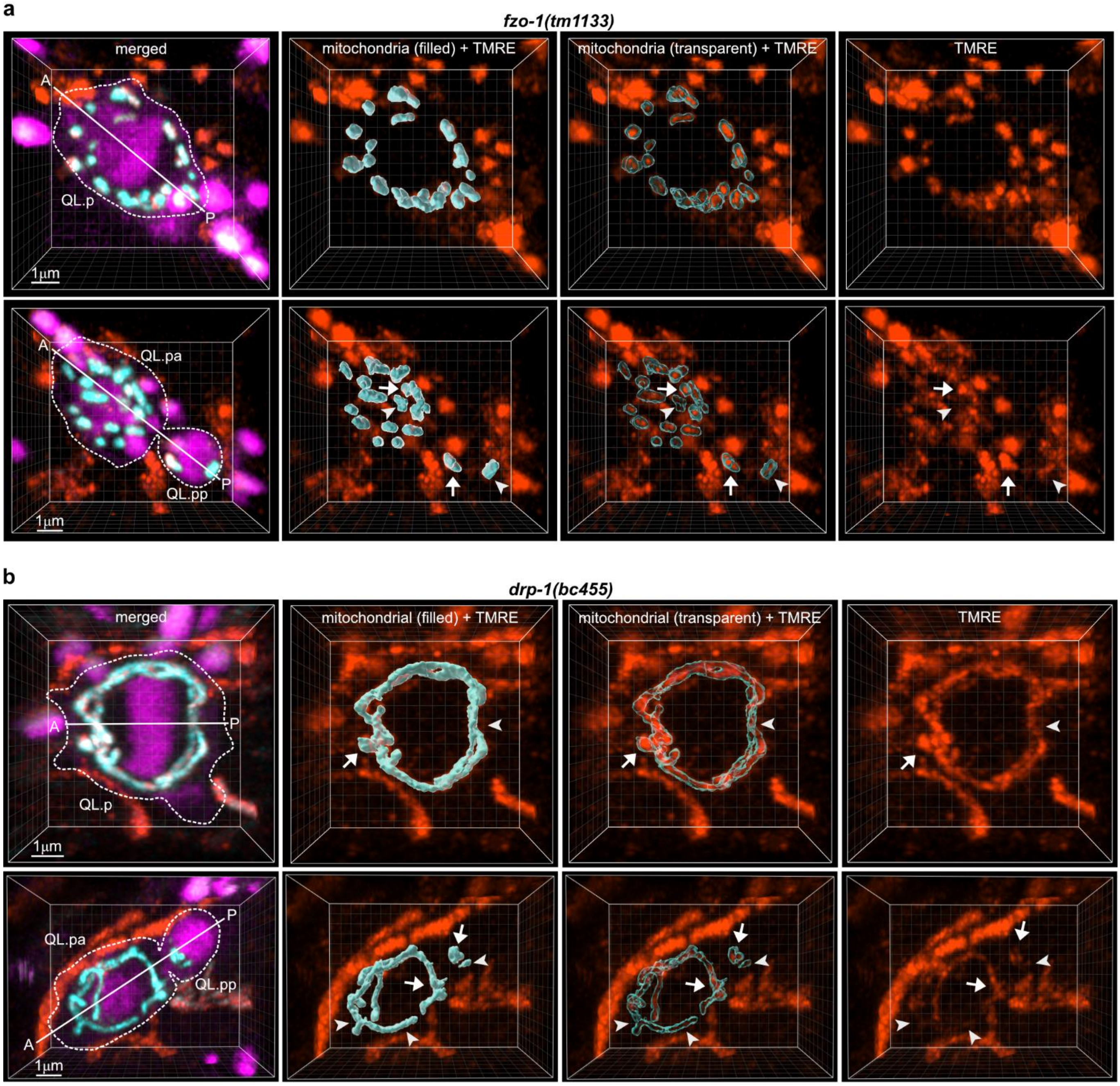


**Figure S13. Mitochondrial activity (TMRE) during QL.p division in *fzo-1(lf)* and *drp-1(lf)* mutants. a**) representative 3D image of *fzo-1(tm1133)* (**a**) and *drp-1(bc455)* (**b**) QL.p cells labelled with TMRE at metaphase (top tow) and post-cytokinesis (bottom row). In both top and bottom left images (merged): both myristoylated SFmTurquoise2ox (cell membrane) and SFmTurquoise2ox::his-24 (chromatin) are shown in magenta, mtGFP (mitochondria) in cyan, and TMRE in orange. The A-P axes show the orientation of QL.p division. Arrows and arrowheads point respectively to mitochondria with higher and lower TMRE intensities. TMRE fluorescence intensities are measured within each volume of mitochondria (see "mitochondria (3D) + TMRE" images) in Imaris. For both genotypes, animals express the *bcIs158* transgene.

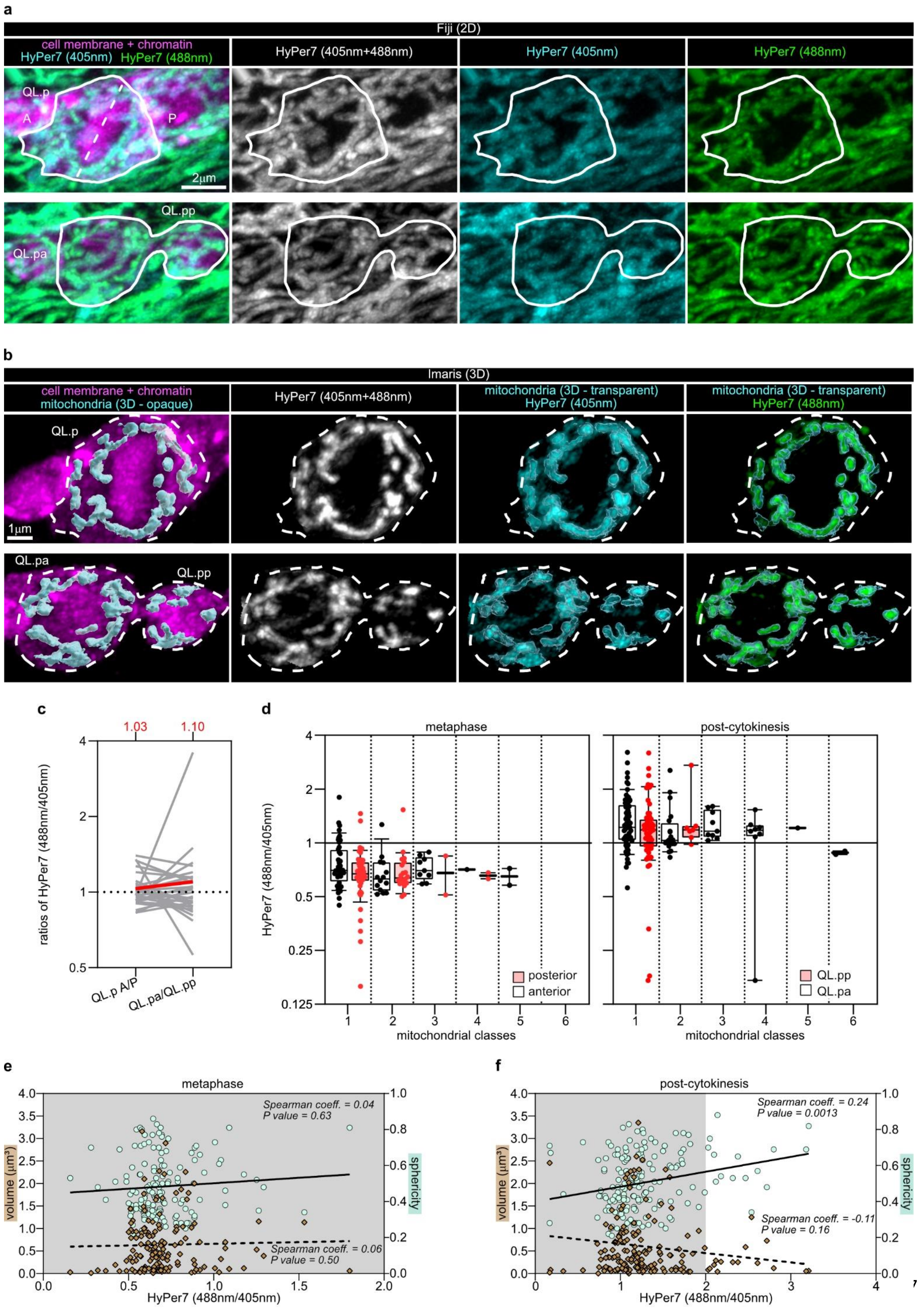
a
Fiji (2D)
cell membrane + chromatin
HyPer7 (405nm) HyPer7 (488nm)
HyPer7 (405nm+488nm)
HyPer7 (405nm)
HyPer7 (488nm)
QL.p
A
P
2μm
QL.pp
QL.pa
b
Imaris (3D)
cell membrane + chromatin
mitochondria (3D - opaque)
HyPer7 (405nm+488nm)
mitochondria (3D - transparent)
HyPer7 (405nm)
mitochondria (3D - transparent)
HyPer7 (488nm)
QL.p
1μm
QL.pa
QL.pp
c
1.03
1.10
ratios of HyPer7 (488nm/405nm)
QL.p A/P
QL.pa/QL.pp
d
metaphase
post-cytokinesis
HyPer7 (488nm/405nm)
posterior
anterior
QL.pp
QL.pa
mitochondrial classes
e
metaphase
Spearman coeff. = 0.04
P value = 0.63
Spearman coeff. = 0.06
P value = 0.50
volume (μm³)
sphericity
HyPer7 (488nm/405nm)
f
post-cytokinesis
Spearman coeff. = 0.24
P value = 0.0013
Spearman coeff. = -0.11
P value = 0.16
volume (μm³)
sphericity
HyPer7 (488nm/405nm)

**Figure S14. Mitochondrial redox state does not determine unequal mitochondrial segregation during QL.p division. a**) Representative 2D image of QL.p at metaphase (top row) and post-cytokinesis (bottom row) in wild-type (+/+) animal expressing mitochondrial HyPer7. Top and bottom left images (merged): both myristoylated mCherry (cell membrane) and mCherry::his-24 (chromatin) are shown in magenta, mitochondrial HyPer7 (violet 405nm excitation) and HyPer7 (green 488nm excitation) are shown in cyan and green, respectively. Metaphase and post-cytokinesis images are representative of 28 divisions imaged from 28 animals. **b**) Same images as in panel **a**, but 3D rendered for mitochondria (cyan in the left panels) using Imaris. **c**) Comparison of the mean ratiometric mitochondrial HyPer7 fluorescence intensity ratio at metaphase (QL.p A/P) and post-cytokinesis (QL.pa/QL.pp) in wild type (W = -22.00 p = 0.8139). **d**) Box and whisker plots of ratiometric mitochondrial HyPer7 fluorescence intensity values per mitochondrion at metaphase (left) and post-cytokinesis (right) in wild type. Boxes represent the interquartile range (IQR; Q1 to Q3; i.e. 50% of data) around the median (line). Upper and lower whiskers represent the 90 and 10 percentiles, respectively. Mitochondria were sorted into six volume classes to investigate the difference in mitochondrial redox between organelles with different volume (see Methods for further information). Wild type metaphase class1 P-class1 A mean diff. = 0.09608 ± 0.04484 SE (F(139) = 4.588) p = 0.0339, class2 P-class2 A mean diff. = -0.0250 ± 0.0770 SE (F(139) = 0.1057) p = 0.7455, class3 P-class3 A mean diff. = 0.04844 ± 0.1726 SE (F(139) = 0.0788) p = 0.7794, class4 P-class4 A mean diff. = 0.0544 ± 0.2704 SE (F(139) = 0.0406) p = 0.8407; Wild type post-cytokinesis (H = 4.377) class1 P-class1 A mean rank diff. = 11.83 p = 0.1356, class2 P-class2 A mean rank diff. = -17.90 p = 0.3575. **e, f**) Correlation between ratiometric mitochondrial HyPer7 fluorescence intensity values and mitochondrial sphericity and volume at single mitochondrion level at metaphase (**e**) and post-cytokinesis (**f**), in wild type. The grey panel in **f** refers to the XY dimensions of in **e**. Continuous line= linear regression of the HyPer7-mitochondrial sphericity correlation; dotted lines= linear regression of HyPer7-mitochondrial volume correlation. P values are calculated using the Wilcoxon matched pairs signed rank test (**c**), the ordinary one-way ANOVA (**d**, left) and the Kruskal Wallis test (**d**, right), both with FDR correction. Normality was tested with the Shapiro-Wilk test. *: P value ≤ 0.05; **: P value ≤ 0.01; ***: P value ≤ 0.001; ****: P value ≤ 0.0001. In panel **c**, the red line and values represent the population average. In panel **e**, dots represent the individual mitochondria. All data are from animals expressing the transgenes *rdvIs1* and *bcIs184*. n= 28. Source data are provided as a Source Data file.

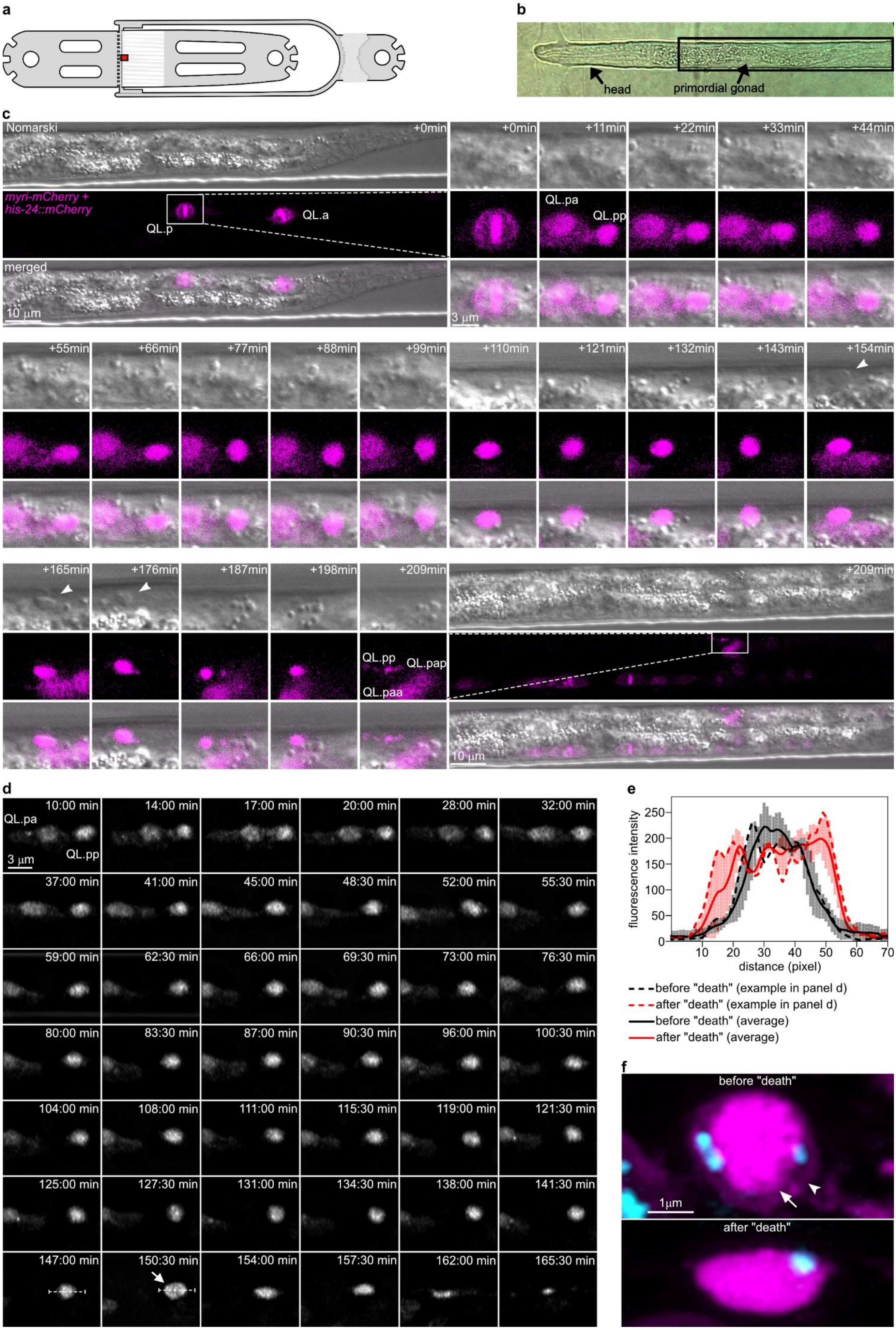
a
b
head
primordial gonad
c
Nomarski
myri-mCherry + his-24::mCherry
QL.p
QL.a
QL.pa
QL.pp
merged
10 µm
3 µm
+0min
+11min
+22min
+33min
+44min
+55min
+66min
+77min
+88min
+99min
+110min
+121min
+132min
+143min
+154min
+165min
+176min
+187min
+198min
+209min
QL.pap
QL.paa
d
3 µm
e
fluorescence intensity
distance (pixel)
before "death" (example in panel d)
after "death" (example in panel d)
before "death" (average)
after "death" (average)
f
before "death"
after "death"
1µm

**Figure S15. Determination of QL.pp survival time.** a) The schematics of the microfluidic device used in this study is the same described in Berger et al. (2021). The red box refers to b. b) L1 larva correctly trapped in a microfluidics channel. The rectangular highlight represents the field of view used for both Nomarski + LSM confocal imaging (c) and Airyscan multiplex confocal imaging (d). c) Nomarski and LSM microscopy of QL.p lineage. The top left and bottom right images represent the enlarged fields of view referring to the first and last time point of the timeseries, respectively. For all images, top, center and bottom represent Nomarski microscopy, confocal microscopy, and merged images. Anterior and posterior are on the left- and right-hand sides, respectively. Between time points +11 and +264, the time-series shows alternate images of the original time series. White arrowheads indicate the refractile appearance of the cell corpse. d) Airyscan multiplex confocal recording of the QL.pp cell death process. The white arrow indicates the first appearance of QL.pp as an elliptical object, while the dotted line is the segment of the image used to construct the fluorescence profiles given in e. e) Fluorescence intensity profiles of QL.pp mCherry chromatin and cell membrane markers. The continuous trends represent the average of 4 cells, while error bars represent the standard deviation. f) Airyscan Super Resolution images of QL.pp before (top) and after (bottom) the morphological change used as the cell death time point in this study (anterior and posterior are left and right, respectively). Before “death”, QL.pp is round and its cell membrane (arrowhead) and nucleus (arrow) are distinguishable, whereas after “death” the nucleus is disintegrated and the release of mCherry::his-24 homogenizes the fluorescence intensity throughout the whole corpse. During “death”, the QL.pp round shape turns into a distinct ellipsoid having the longer axes always oriented along the anterior-posterior axes of the animal. In this figure, animals express the bcIs153 transgene. Source data are provided as a Source Data file.

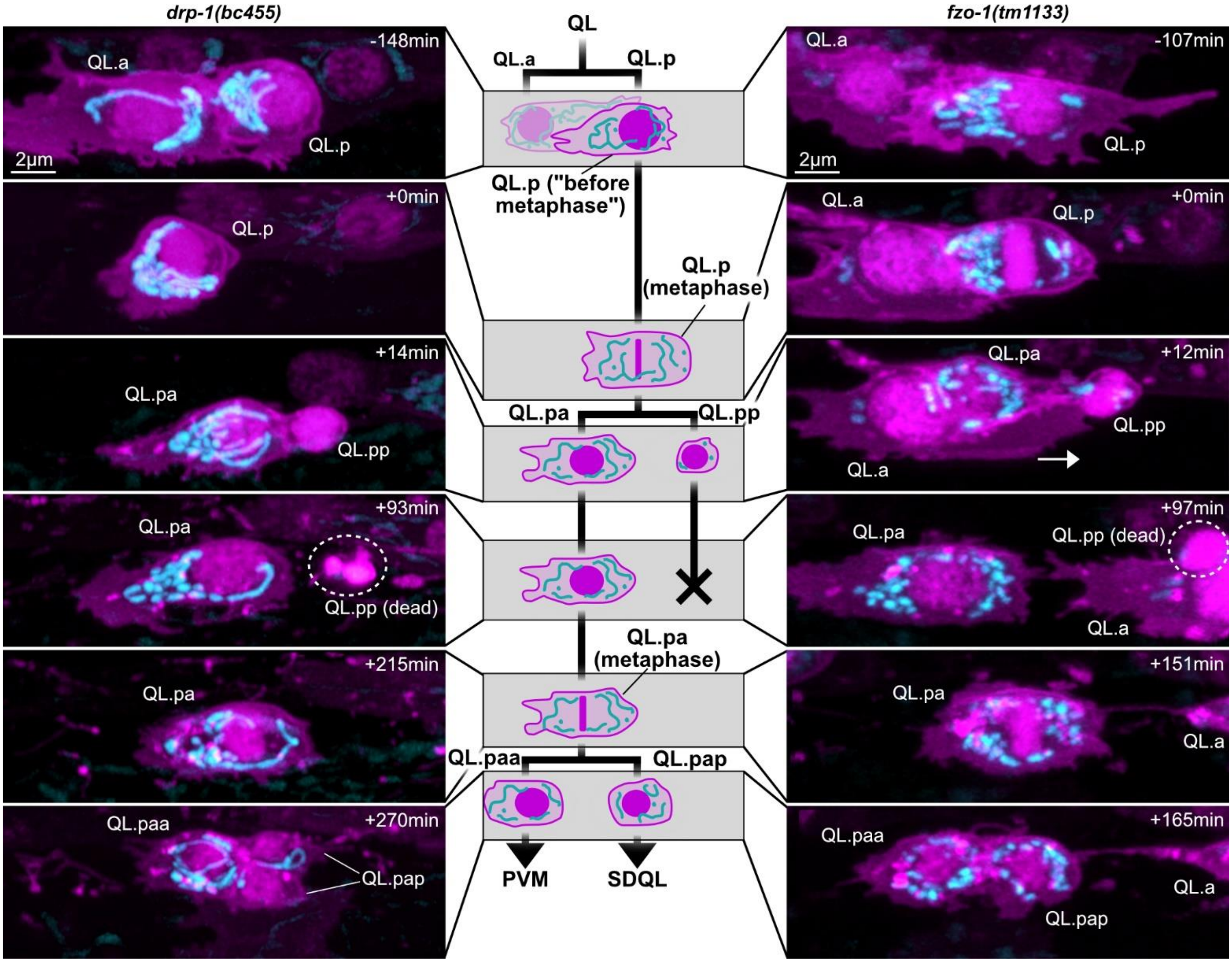


**Figure S16. In the absence of mitochondrial dynamics, QL.pp can inherit few or no mitochondria.** Super-resolution live two-colour images of QL.p lineages. Plasma membrane (myristoylated mCherry) and chromatin (mCherry::his-24) are showed in magenta, mitochondria (mtGFP) in cyan (*bcIs153* transgene). Images are maximum intensity projections of aligned z-stacks. Both image columns represent examples of abnormal QL.p mitochondrial partitioning in *drp-1(bc455)* and *fzo-1(tm1133)* (left and right, respectively) that caused QL.pp to inherit no mitochondria (left, time +14) or few mitochondria (right, time +12). In both cases, QL.pp died relatively fast (dotted circles) (+93 and +97 for *drp-1(bc455)* and *fzo-1(tm1133)*, respectively) (compare with QL.pp death time ranges in Fig.4 e and g). All images refer to the relative time point depicted along the schematics of the QL.p lineage shown in the centre. L1 larvae developed in the microfluidics device at high resolution and images were taken in Airyscan super resolution. Images were taken in super resolution even before QL.p division and during the disappearance of QL.pp cells in these abnormal cases for illustration (see also the abnormal distribution of mitochondria in both QL.p examples soon after QL division). The time scale is centred to metaphase (time +0min).
