# Supplementary material for: Unequal segregation of mitochondria during asymmetric cell division contributes to cell fate divergence in sister cells in vivo
Source: Nat Commun. 2025 Aug 4;16:7174. doi: 10.1038/s41467-025-62484-5 (PMC12322191; doi:10.1038/s41467-025-62484-5)
Supplement: Supplementary file 3 — Reporting Summary [file 41467_2025_62484_MOESM3_ESM.pdf]

nature portfolio

Corresponding author(s): Barbara Conradt

Last updated by author(s): Jul 6, 2025

Reporting Summary

Nature Portfolio wishes to improve the reproducibility of the work that we publish. This form provides structure for consistency and transparency in reporting. For further information on Nature Portfolio policies, see our Editorial Policies and the Editorial Policy Checklist.

Statistics

For all statistical analyses, confirm that the following items are present in the figure legend, table legend, main text, or Methods section.

n/a	Confirmed	
☐	☒	The exact sample size (*n*) for each experimental group/condition, given as a discrete number and unit of measurement
☐	☒	A statement on whether measurements were taken from distinct samples or whether the same sample was measured repeatedly
☐	☒	The statistical test(s) used AND whether they are one- or two-sided<br>*Only common tests should be described solely by name; describe more complex techniques in the Methods section.*
☐	☒	A description of all covariates tested
☐	☒	A description of any assumptions or corrections, such as tests of normality and adjustment for multiple comparisons
☐	☒	A full description of the statistical parameters including central tendency (e.g. means) or other basic estimates (e.g. regression coefficient) AND variation (e.g. standard deviation) or associated estimates of uncertainty (e.g. confidence intervals)
☐	☒	For null hypothesis testing, the test statistic (e.g. *F*, *t*, *r*) with confidence intervals, effect sizes, degrees of freedom and *P* value noted<br>*Give P values as exact values whenever suitable.*
☒	☐	For Bayesian analysis, information on the choice of priors and Markov chain Monte Carlo settings
☒	☐	For hierarchical and complex designs, identification of the appropriate level for tests and full reporting of outcomes
☐	☒	Estimates of effect sizes (e.g. Cohen's *d*, Pearson's *r*), indicating how they were calculated

Our web collection on statistics for biologists contains articles on many of the points above.

Software and code

Policy information about availability of computer code

Data collection	ZenBlue 3.3, Imaris 10.2.0, Fiji v1.54p
Data analysis	GraphPad Prism8 and R (v4.3.2)

For manuscripts utilizing custom algorithms or software that are central to the research but not yet described in published literature, software must be made available to editors and reviewers. We strongly encourage code deposition in a community repository (e.g. GitHub). See the Nature Portfolio guidelines for submitting code & software for further information.

Data

Policy information about availability of data

All manuscripts must include a data availability statement. This statement should provide the following information, where applicable:

- Accession codes, unique identifiers, or web links for publicly available datasets
- A description of any restrictions on data availability
- For clinical datasets or third party data, please ensure that the statement adheres to our policy

Data will be made available as supplementary.

Research involving human participants, their data, or biological material

Policy information about studies with human participants or human data. See also policy information about sex, gender (identity/presentation), and sexual orientation and race, ethnicity and racism.

Reporting on sex and gender	NA
Reporting on race, ethnicity, or other socially relevant groupings	NA
Population characteristics	NA
Recruitment	NA
Ethics oversight	NA

Note that full information on the approval of the study protocol must also be provided in the manuscript.

Field-specific reporting

Please select the one below that is the best fit for your research. If you are not sure, read the appropriate sections before making your selection.

[x] Life sciences   [ ] Behavioural & social sciences   [ ] Ecological, evolutionary & environmental sciences

For a reference copy of the document with all sections, see nature.com/documents/nr-reporting-summary-flat.pdf

Life sciences study design

All studies must disclose on these points even when the disclosure is negative.

Sample size	C elegans has invariant cell lineages. Sample sizes for super resolution imaging were generally set around 30 where the central limit theory generally holds. Biological relevance and significance were deemed more important, and followed up for the central tenets of the paper by checking for survival of the daughter cells.
Data exclusions	Data were only excluded where values produced infinites (or NAs depending on software) through dividing by zero. (I.e. rare situations where smaller daughter cells inherited no mitochondria, or drp-1 spots; these are indicated in the text.)
Replication	The core tenets of the paper were replicated through imaging using microbeads, and through imaging using microfluidics. Both methods came to the same conclusions. Different reporter strains (bcIs153 and bcIs158) and staining methods (TMRE) all corroborated the same finding that mitochondria were asymmetrically inherited and are all reported in the paper.
Randomization	Randomisation was not relevant to this study, as genotypes are unambiguous, and relevant controls were reported.
Blinding	Blinding was not relevant to the study, as genotypes are unambiguous, no subjective measures were taken, and variables were quantified using standardised means (Imaris).

Reporting for specific materials, systems and methods

We require information from authors about some types of materials, experimental systems and methods used in many studies. Here, indicate whether each material, system or method listed is relevant to your study. If you are not sure if a list item applies to your research, read the appropriate section before selecting a response.

Materials & experimental systems

n/a	Involved in the study	
[x]	[ ]	Antibodies
[x]	[ ]	Eukaryotic cell lines
[x]	[ ]	Palaeontology and archaeology
[ ]	[x]	Animals and other organisms
[x]	[ ]	Clinical data
[x]	[ ]	Dual use research of concern
[x]	[ ]	Plants

Methods

n/a	Involved in the study	
[x]	[ ]	ChIP-seq
[x]	[ ]	Flow cytometry
[x]	[ ]	MRI-based neuroimaging

Animals and other research organisms

Policy information about studies involving animals; ARRIVE guidelines recommended for reporting animal research, and Sex and Gender in Research

Laboratory animals	Caenorhabditis elegans, Bristol N2 strain
Wild animals	The study did not involve wild animals.
Reporting on sex	All animals used were hermaphrodite. Sex was not considered in the study, as including males in C elegans studies generally implies including another knockout mutation (e.g. a High Incidence of Males (increased X chromosome loss) mutant) which may have knockon effects. Additionally, sex was not deemed relevant to the subcellular nature of the study (organelle inheritance in asymmetrically dividing neuroblasts).
Field-collected samples	The study did not involve samples collected from the field.
Ethics oversight	No ethical approval or guidance was required: the model organism C elegans is generally considered free from ethical concern.

Note that full information on the approval of the study protocol must also be provided in the manuscript.

Plants

Seed stocks	No seeds or plants were used in this study.
Novel plant genotypes	No seeds or plants were used in this study.
Authentication	No seeds or plants were used in this study.
